# The role of socio-economic disadvantage in the development of comorbid emotional and conduct problems in children with ADHD

**DOI:** 10.1007/s00787-017-0940-z

**Published:** 2017-01-07

**Authors:** Eirini Flouri, Emily Midouhas, Alexandra Ruddy, Vanessa Moulton

**Affiliations:** Department of Psychology and Human Development, UCL Institute of Education, University College London, 25 Woburn Square, London, WC1H 0AA UK

**Keywords:** ADHD, Comorbidity, Conduct problems, Emotional problems, Parenting, Socio-economic disadvantage

## Abstract

Previous research shows that, compared to children without ADHD, children with ADHD have worse socio-emotional outcomes and more experience of socio-economic disadvantage. In this study, we explored if and how the increased emotional and behavioural difficulties faced by children with ADHD may be accounted for by their more disadvantaged socio-economic circumstances. Our study, using data from 180 children (149 boys) with ADHD from the Millennium Cohort Study, had two aims. First, to examine the role of socio-economic disadvantage in the trajectories of emotional and conduct problems in children with ADHD at ages 3, 5, 7 and 11 years. Second, to explore the roles of the home environment (household chaos) and parenting (quality of emotional support, quality of the parent–child relationship and harsh parental discipline) in mediating any associations between socio-economic disadvantage and child emotional and conduct problems. Using growth curve models, we found that socio-economic disadvantage was associated with emotional and conduct problems but neither the home environment nor parenting attenuated this association. Lower quality of the parent–child relationship and harsher discipline were associated with more conduct problems. It appears that socio-economic disadvantage and parenting contribute independently to the prediction of comorbid psychopathology in children with ADHD.

**Electronic supplementary material:**

The online version of this article (doi:10.1007/s00787-017-0940-z) contains supplementary material, which is available to authorized users.

## Introduction

Children with ADHD are at increased risk of emotional and behavioural problems and disorders [1–3]. Comorbidity rates for ADHD and internalising problems, including anxiety and depression, range from 10 to 60% [3–5] and those for ADHD and externalising disorders, such as oppositional defiant disorder (ODD) and conduct disorder (CD), range from 20 to 60% [6, 7]. ADHD accompanied by comorbid emotional and behavioural problems can lead to or exacerbate learning difficulties, antisocial behaviour, other psychopathologies and poor well-being [7].

Numerous studies have confirmed the heritability of ADHD [8–10]. However, environmental factors can also impact on both the development of ADHD and its associated impairments [11, 12]. For example, family disruption, harsh or disengaged parenting and parental depression are more likely to be experienced by children and adolescents with ADHD and comorbid psychiatric conditions than those with ADHD alone [13]. Family socio-economic disadvantage may be another factor associated with such comorbidity. Socio-economic disadvantage is a powerful antecedent of emotional and behavioural problems in both typically [14] and atypically developing children [15], and a risk factor of ADHD [12, 16].

Nonetheless, only few studies have investigated its role in psychiatric comorbidity with ADHD [13, 17–19]. For example, Larson et al. [19] showed that socio-economic disadvantage is associated with a greater likelihood of multiple comorbidities in ADHD, and Hurtig et al. [13] that it is more common among children and adolescents with ADHD and comorbid psychiatric conditions than among those with ADHD alone. However, none of these studies explored the role of socio-economic disadvantage or socio-economic status (SES) in the development (trajectories) of comorbid emotional and behavioural problems among children with ADHD. Experiencing chronic socio-economic disadvantage could result in children with ADHD showing increasingly worse adjustment. By exploring the association of socio-economic disadvantage with the level and growth of emotional and behavioural problems in children with ADHD, this study’s first aim was to fill this gap. Its second aim was to attempt to explain this expected association. Much evidence has been accumulated to suggest that socio-economic disadvantage impacts on child mental health adversely (the social causation thesis in child psychopathology) through both increasing family stress and reducing family investments in children [20–22]. Thus, the impact of socio-economic disadvantage on the course of ADHD children’s emotional/behavioural development may be due, at least partly, to their home environment—insofar as this results from, or reflects, parental stress—and the parenting they receive. Drawing on this research, this study explored the roles of parenting styles and the home environment in explaining any associations between socio-economic disadvantage and level and development of emotional and behavioural problems among children with ADHD.

### Parenting and emotional and behavioural problems in children with ADHD

Parenting and the home environment are associated with emotional and behavioural problems both in children with ADHD and in those without. For example, a well-researched aspect of the home environment, household chaos, defined as “high levels of ambient stimulation (e.g. noise, overcrowding), minimal structure and routine, and considerable unpredictability and confusion in daily activities” [23, p. 561], is more common in low-income households [24] and is related to both emotional/behavioural problems [14, 25] and hyperactivity/poor attention regulation in children [26, 27]. Parenting styles also predict comorbid psychopathology, especially conduct problems, in children with ADHD. For example, Chronis et al. [28] found that mothers’ positive (i.e. praise and positive affect) but not negative parenting predicted the developmental course of conduct problems in children with ADHD. Other research has found that mothers of children with ADHD and comorbid CD/ODD report higher levels of perceived child-rearing hassles and rejecting parenting [29] and are more likely to be negative, directive and rejecting, compared to mothers of typically developing children or children with non-comorbid ADHD [11]. Some research has also described the parenting of families with children with ADHD and comorbid internalising problems. For example, compared to mothers of ADHD children without comorbid anxiety, mothers of children with ADHD and comorbid anxiety are more possessive and practice less positive parenting [3]. Similarly, the family environments of anxious ADHD children tend to be more controlling, less encouraging of independence and less self-sufficient than those of non-anxious ADHD children and non-ADHD children [30].

### The present study

The research findings reviewed above suggest that, when compared with non-ADHD children, children with ADHD have more emotional and conduct problems and are more likely to be exposed to socio-economic disadvantage during childhood. Given that exposure to socio-economic disadvantage has been linked to emotional and conduct problems in children, we attempted in this study to estimate the extent to which the increased emotional and conduct problems faced by children with ADHD may be accounted for by their more disadvantaged socio-economic circumstances. Drawing on findings from research with typically developing children, we also attempted to investigate if any associations between socio-economic disadvantage and these difficulties may be explained by parenting styles and the home environment. To answer these two research questions, we used longitudinal data from the UK’s Millennium Cohort Study (MCS), collected when children were 3, 5, 7 and 11 years old.

### Participants and procedure

MCS is a population-based longitudinal birth cohort study of children born in the UK over 12 months from 1 September 2000. Children were around 9 months old at Sweep 1, and around 3, 5, 7 and 11 years old at Sweeps 2, 3, 4 and 5, respectively. MCS was designed to over-represent families living in areas of high child poverty, areas with high proportions of ethnic minority populations across England, and the three smaller UK countries. Parent-reported data were collected through interviews and self-completion questionnaires. Ethical approval was gained from NHS Multi-Centre Ethics Committees, and parents gave informed consent before interviews took place. At Sweep 1, 18,522 families participated in MCS. The numbers of productive families at Sweeps 2, 3, 4 and 5 were 15,590, 15,246, 13,857 and 13,287, respectively.

The current study included only singleton children and the first-born child of the families with twins or triplets in the cohort. The analytic sample (i.e. the ‘ADHD’ sample; *n* = 180, 149 males) consisted of children with a parent-reported medical diagnosis of ADHD at Sweep 4, when children were aged 7 years. Diagnosis was based on the primary caregiver’s answer to the question “Has a doctor or health professional ever told you that (Cohort child’s name) had ADHD?” The primary caregivers of 180 (1.3%) children responded ‘yes’ to this question. The ‘non-ADHD’ sample (*n* = 13,568, 6822 males) comprised children whose primary caregivers responded ‘no’ to this question (109 families refused to answer or responded “don’t know”/“not applicable”). The non-ADHD sample was used only for descriptive comparisons in this study.

### Measures

*Emotional and conduct problems* were measured with the emotional symptoms and conduct problems subscales of the parent-reported Strengths and Difficulties Questionnaire (SDQ) [33]. The SDQ was completed by the main caregivers at Sweeps 2–4 (ages 3–7) and both the main caregivers and their partners at Sweep 5 (age 11). In view of the small size of the analytic sample, the partner-reported SDQ scores were used when the main caregiver’s SDQ data were missing. The emotional symptoms and conduct problems subscales have five items each. Each item is rated on a 3-point scale from *not true* (0) to *certainly true* (2). The emotional symptoms subscale had good internal consistency at Sweeps 4 and 5 (*α* = 0.72 and *α* = 0.77, respectively), but poor at Sweeps 2 and 3 (*α* = 0.56 and *α* = 0.58, respectively). The conduct problems subscale had good internal consistency at Sweep 2 (*α* = 0.70) and Sweep 5 (*α* = 0.75), and acceptable at Sweeps 3 (*α* = 0.68) and 4 (*α* = 0.68).

*Family socio*-*economic disadvantage* was measured at Sweeps 2–5 with a composite measure [34]. This was the mean of four dichotomous variables: overcrowding (more than 1.5 people per room excluding the bathroom and kitchen), lack of home ownership, receipt of income support, and income poverty (below the poverty line).

The home environment in MCS at Sweep 2 was assessed by responses on three 5-point items, completed by the parent, from the Confusion, Hubbub, and Order Scale (CHAOS) [35]: “It’s really disorganised in our home”, “You can’t hear yourself think in our home” and “The atmosphere in our home is calm” (reverse-coded; *α* = 0.74). CHAOS is a good and widely used measure of *household chaos* and captures a broad construct of chaotic living conditions, characterised not only by factors such as noise and crowding, but also by qualities such as lack of structure and routine.

Parenting was assessed by *quality of emotional support, quality of the parent*–*child relationship* and *harsh parental discipline* at Sweep 2. Eight items from the Emotional Support subscale of the Home Observation and Measurement of the Environment-Short Form [36], completed by an interviewer, were used to measure the quality of emotional support (*α* = 0.63). Items included “Mother answers child’s questions or requests verbally” and “Mother spontaneously praises child’s qualities or behaviour twice during the visit”. Quality of the parent–child relationship was measured with the mother’s report on the short form of the Child-Parent Relationship Scale [37] comprising 15 5-point items (*α* = 0.79). Straus’s Conflict Tactics Scale for Parent and Child [38], completed by the parent, was used to measure the frequency of seven parental discipline practices (on 5-point scales), such as shouting at or smacking the child (*α* = 0.67).

Finally, we controlled for covariates associated with both socio-economic disadvantage and emotional/conduct problems among children with ADHD [13], including *maternal education* (university degree or not), *family structure* (two caregivers at home or not; measured at Sweeps 2–5) and *maternal psychological distress*, measured at Sweeps 2–5 with the K6 [39] (*α* = 0.87–0.91 across sweeps). Our child-level covariates were *cognitive ability* and *low birthweight* (<2.5 kilos), also in view of their strong associations with both socio-economic disadvantage [14] and psychiatric comorbidity in ADHD [31, 32], as well as *gender* and *ethnicity*. In MCS, cognitive ability was measured differently at different ages, and so we used the age 3 (Sweep 2) measures to capture its role at the beginning of the trajectories of conduct and emotional problems. At Sweep 2, the ability measures were the British Ability Scales II (BAS II) Naming Vocabulary subtest and the Bracken School Readiness Assessment-Revised (BSRA-R). BAS Naming Vocabulary measures the child’s expressive language, vocabulary and general language development. The BSRA-R assesses knowledge and understanding of basic concepts relating to colours, letters, numbers/counting, sizes, comparisons and shapes. Ethnicity, categorised into six groups (White, Mixed, Indian, Pakistani/Bangladeshi, Black and Other) was collapsed into two (white or not) given the small sample size.

### Data analysis plan

We fitted growth curve models [40] to examine the direct ‘effect’ of socio-economic disadvantage, and its mediation via parenting and the home environment, on the level and development of ADHD children’s comorbid emotional and conduct problems at ages 3–11 years. This approach allowed the estimation of individual problem trajectories by specifying an independent variable for time. In this study, the time variable was age in years, grand mean-centred at age 7. We had a random slope for age and for age^2^ to describe individual linear trajectories and to allow for non-linear trajectories (see “Descriptive analysis”). Our models were two-level, with occasion (Level 1) nested within child (Level 2). This approach not only captures the individual differences between children’s emotional and conduct problems at different ages but also takes into account the correlations of these measures over time for each child. Our models also specified fixed parameters. These are the intercept (the mean emotional and conduct problem scores at age 7) and the linear slope (the mean change in scores per year). As explained in the Descriptive Analysis section, emotional problems had a quadratic trajectory, on average, and conduct problems a cubic, on average (described below). Therefore, we also included a fixed effect for age^2^ (to capture the non-constant average rate of annual change) for conduct and emotional problems, and for age^3^ for conduct problems. We did not have enough time-points of data to include a random effect for age^3^. (The random parameters and what they show are described in detail in the Supplement).

Models were estimated in *MLwiN 2.33*, a statistical package for multilevel modelling.

The sequence of models fitted are shown in Table 1. Model 1 contained age and age^2^ entered as fixed and random effects. Age^3^ was also entered as a fixed effect in the models for conduct problems, as explained. Models 2–4 were conditional, and therefore, the variances and covariances reflect residual variability, i.e. variability not accounted for by the considered covariates. In view of our small sample size, we acknowledge that the results of the more complex models, such as Models 3 and 4 must be treated with caution. The MCS oversampling of families from areas (wards) of high child poverty, high proportions of ethnic minorities (in England) and the smaller UK countries was accounted for in all conditional models by controlling for stratification.^1^

**Table 1:** Model summary

Models	Specification
Model 1	Age (centred at 7) in years + age^2^ + age^3a^
Model 2	Model 1 + stratum variables^b^ + family SED + family SED × age + family SED × age^2^ + family SED × age^3c^
Model 3	Model 2 + family^d^ covariates + child^e^ covariates
Model 4	Model 3 + parent–child relationship + harsh parental discipline + quality of emotional support + household chaos

*SED* socio-economic disadvantage

^a,c^For conduct problems only

^b^The MCS stratum variables are England-advantaged (reference group), England-disadvantaged, England-ethnic, Wales-advantaged, Wales-disadvantaged, Scotland-advantaged, Scotland-disadvantaged, Northern Ireland-advantaged, and Northern Ireland-disadvantaged

^d^Maternal psychological distress, maternal education, and family structure

^e^Gender, ethnicity, low birthweight, and cognitive ability

## Results

### Descriptive analysis

Table 2 shows that the families of children with ADHD experienced more socio-economic disadvantage than those of non-ADHD children. At baseline (age 3 years), the mothers of children with ADHD reported poorer parent–child relationships and more use of harsh discipline, compared to the mothers of non-ADHD children. Moreover, children with ADHD experienced lower quality of emotional support and more household chaos, compared to children without ADHD. The study variables were weakly or moderately inter-related. For example, correlations among the four parenting and home environment variables ranged from −0.09 (for the association between harsh parental discipline and quality of emotional support) to 0.32 (for that between quality of emotional support and quality of the parent–child relationship). Concurrent correlations between socio-economic disadvantage and the parenting and home environment variables were also weak or moderate at −0.29 (quality of the parent–child relationship), −0.20 (quality of emotional support), −0.05 (harsh parental discipline) and 0.09 (household chaos).

**Table 2:** Key predictors of comorbid emotional and conduct problems at age 3 (baseline) for the ADHD and non-ADHD samples (unweighted data)

Variable	ADHD (*N* = 180)			Non-ADHD (*N* = 13,568)				
	*n*	*M*	SD	*n*	*M*	SD	*t*	*df*
Family SED	153	0.36	0.33	12,342	0.21	0.29	5.44*	154.87
Parent–child relationship	136	58.18	8.10	10,986	64.35	6.93	−8.84*	137.46
Harsh parental discipline	131	3.28	0.79	10,976	2.85	0.72	6.81*	11,105
Quality of emotional support	146	1.89	0.17	11,597	1.95	0.11	−4.26*	146.59
Household chaos	153	2.66	0.85	12,342	2.33	0.72	4.81*	154.73

*SED* socio-economic disadvantage

* *p* < .01

**Fig. 1:**
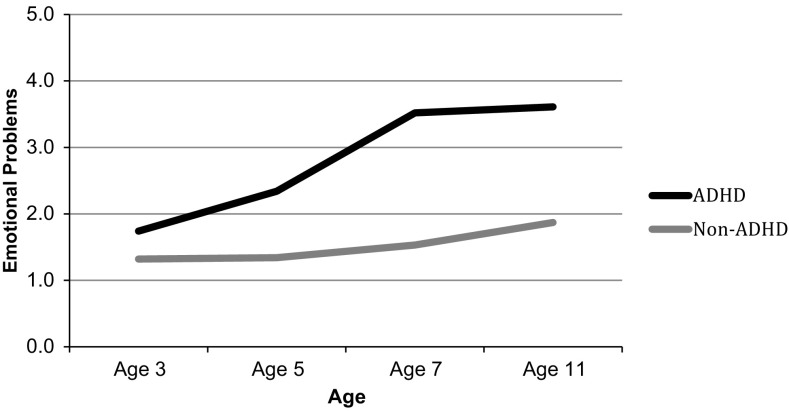
Weighted mean trajectory of emotional problems of ADHD and non-ADHD children

**Fig. 2:**
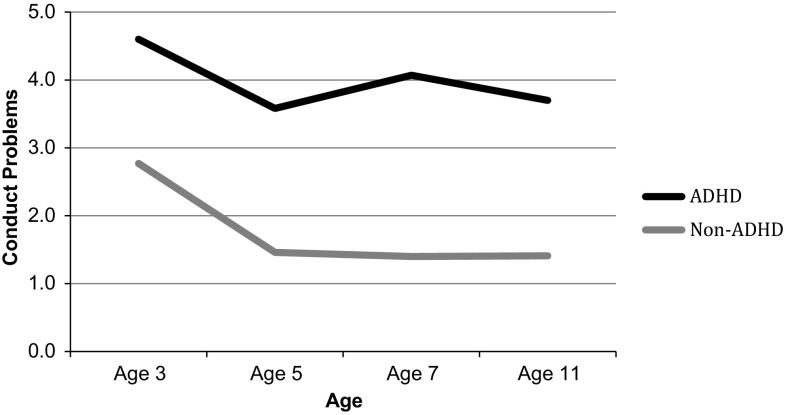
Weighted mean trajectory of conduct problems of ADHD and non-ADHD children

As expected, children with ADHD had higher levels of both emotional and conduct problems than non-ADHD children across the entire study period (Figs. 1, 2). As can be seen, the mean trajectory for ADHD children’s emotional symptoms had a non-linear shape, increasing steadily between ages 3 and 7 years before reaching a plateau between the ages of 7 and 11. The non-ADHD sample, on the other hand, had a mean emotional symptoms score that was unchanging between ages 3 and 5 years, before increasing slightly and steadily between the ages 5 and 11. As for conduct problems, the mean score of the ADHD sample decreased from ages 3–5 years, and then increased until age 7 before decreasing again between ages 7 and 11. After decreasing between the ages of 3 and 5 years, the conduct problems of children in the non-ADHD group levelled off.

### Growth curve models

As presented in Table 3 (Model 1), emotional and conduct problem scores increased annually by 0.267 (SE = 0.029) and 0.257 (SE = 0.078), respectively. The effect of age^2^ was significant on emotional symptoms and that of age^3^ was significant on conduct problems, reflecting the quadratic and cubic shapes of the average trajectories for emotional and conduct problems, respectively, identified in the descriptive analysis. As can be seen, the between and within-child variation in problems was significant, as were the intercept-slope variances covariances for both age and age^2^ for both problem types. This suggests, respectively, that emotional and conduct problem scores varied significantly both between children and within children over time, and that the level of the age 7 problem score was related to the rate of change in scores over time.

**Table 3:** Fixed and random effects on emotional and conduct problems

Predictors	Model 1				Model 4			
	Emotional problems		Conduct problems		Emotional problems		Conduct problems	
	Coeff.	SE	Coeff.	SE	Coeff.	SE	Coeff.	SE
Fixed effects								
Age	0.267***	0.029	0.257***	0.078	0.294***	0.050	0.286*	0.140
Age^2^	−0.041***	0.010	0.013	0.010	−0.024	0.018	0.041**	0.015
Age^3^			−0.023***	0.005			−0.024**	0.009
England-disadvantaged					−0.172	0.414	−0.241	0.369
England-ethnic					0.964	0.946	0.911	0.832
Wales-Advantaged					0.654	0.717	−0.898	0.635
Wales-disadvantaged					−0.076	0.445	−0.462	0.392
Scotland-advantaged					−0.406	0.704	−0.976	0.614
Scotland-disadvantaged					−1.540**	0.572	−1.097*	0.506
Northern Ireland-advantaged					0.373	0.860	−1.392	0.784
Northern Ireland-disadvantaged					−0.497	0.549	−0.396	0.492
Family SED					1.735**	0.663	1.481*	0.614
Family SED × age					−0.096	0.118	−0.382	0.341
Family SED × age^2^					−0.068	0.043	−0.075*	0.036
Family SED × age^3^							0.019	0.021
Female					0.076	0.384	−0.024	0.345
White					−0.577	0.827	1.283	0.722
Low birthweight					0.299	0.562	0.019	0.495
BAS II naming vocabulary					0.008	0.016	−0.004	0.014
BSRA-R					0.002	0.012	0.004	0.011
Maternal psychological distress					0.108***	0.023	0.090***	0.021
Mother is university-educated					−0.333	0.499	−0.706	0.439
Two caregivers					0.521	0.286	0.348	0.260
Parent-child relationship					−0.031	0.023	−0.105***	0.021
Harsh parental discipline					0.092	0.202	0.360*	0.180
Quality of emotional support					0.357	0.873	0.071	0.772
Household chaos					−0.034	0.181	0.119	0.161
Constant	3.307***	0.178	4.016***	0.171	2.946	2.395	6.180**	2.117
Random effects								
Between-child intercept variance	4.317***	0.621	3.919***	0.554	3.090***	0.644	2.497***	0.529
Between-child slope variance (age)	0.068***	0.017	0.057***	0.015	0.064**	0.021	0.054**	0.017
Between-child intercept slope variance covariance (age)	0.268***	0.075	0.147*	0.064	0.313***	0.087	0.214**	0.070
Between-child slope variance (age^2^)	0.005*	0.002	0.007***	0.002	0.006*	0.003	0.003	0.002
Between-child intercept slope variance covariance (age^2^)	−0.103***	0.029	−0.092***	0.027	−0.091**	0.034	−0.082**	0.028
Between-child slope (age) slope (age^2^) variance covariance	−0.001	0.004	−0.011**	0.004	−0.004	0.005	−0.007	0.004
Between-occasion variance	2.049***	0.226	1.741***	0.194	1.931***	0.279	1.644***	0.234

*SED* socio-economic disadvantage, *BAS II* British Ability Scales II, *BSRA*-*R* Bracken School Readiness Assessment Revised

* *p* < .05; ** *p* < .01; *** *p* < .001

In Model 2 (Supplementary Table 1), there was a main effect of socio-economic disadvantage on age 7 emotional (*b* = 0.919, SE = 0.465) and conduct problems (*b* = 1.404, SE = 0.448). The effect of socio-economic disadvantage on annual change in scores was significant for emotional problems only (*b* = −0.177, SE = 0.090). Model 3 showed that the family and child covariates attenuated the interaction effect of socio-economic disadvantage and age on emotional problems. However, the main effect of socio-economic disadvantage on both emotional and conduct problems at age 7 remained significant (*b* = 1.255, SE = 0.616 and *b* = 1.784, SE = 0.575, respectively). Although the effect of the interaction between socio-economic disadvantage and age (i.e. the effect of socio-economic disadvantage on the average rate of annual change in scores) was not significant on conduct problems, the effect of the interaction between family socio-economic disadvantage and age^2^ was (*b* = −0.082, SE = 0.034). Of the family covariates, there was a significant main effect of maternal psychological distress on emotional (*b* = 0.123, SE = 0.020) and conduct problems (*b* = 0.114, SE = 0.019). No other family or child covariates were significant for emotional problems. For conduct problems, BSRA-R had a negative effect (*b* = −0.026, SE = 0.011).

Model 4 (Table 3) showed that neither parenting nor the home environment mediated the effect of socio-economic disadvantage on emotional or conduct problems at age 7. However, there were two significant effects on conduct problems: a negative main effect of quality of the parent–child relationship and a positive main effect of harsh parental discipline. None of the parenting or the home environment variables had a significant main effect on emotional problems.

## Discussion

Children with ADHD are at high risk of developing comorbid emotional and behavioural problems, which are, in turn, associated with adverse long-term outcomes. Therefore, it is important for these children and their families that research identifies the potentially modifiable factors that predict the development of socio-emotional problems over time. In a UK population sample of children with ADHD followed from ages 3–11 years, we explored the roles of three such factors (socio-economic disadvantage, parenting and the home environment). We characterised parenting by quality of the parent–child relationship, quality of emotional support and harsh parental discipline, and we characterised the home environment by household chaos. As expected, compared to children without ADHD, children with ADHD experienced more socio-economic disadvantage, harsher parenting, poorer relationships with their parents, less emotional support, more household chaos and more emotional and conduct problems. Furthermore, as children with ADHD got older, they developed more emotional problems relative to children without ADHD. Despite differences in the level of conduct problems, both ADHD and non-ADHD children displayed fewer conduct problems after they started school, which is consistent with previous findings [41, 42].

Although previous studies have reported an increased risk of comorbid disorders in ADHD children from low-income or low-SES families [13, 17–19], ours is the first study to explore the association between socio-economic disadvantage and the development (growth) of emotional and conduct problems of children with ADHD. In line with the evidence for the adverse effect of socio-economic disadvantage on emotional and behavioural outcomes in typically developing children [43], we found that children with ADHD from families with higher levels of socio-economic disadvantage had more emotional and conduct problems than their counterparts. Our study, however, showed that family socio-economic disadvantage was not related to children’s trajectories of emotional and conduct problems. We may find that, as children with ADHD move into adolescence, socio-economic disadvantage can alter the course of these problems, via its effects on important environmental influences during this period, such as peers. With additional sweeps of data, we can explore this possibility.

Nonetheless, family socio-economic disadvantage was significantly related to both emotional and conduct problems in our sample and was robust to adjustment for family structure and maternal psychological distress and education. If this effect of socio-economic disadvantage proves to be causal, then efforts to avoid or reduce socio-economic disadvantage should be a policy priority for children with ADHD and their families. However, socio-economic disadvantage was not, as we had hypothesised, explained by parenting or the home environment. Therefore, it did not lead to problem behaviour in children with ADHD because it impaired the parenting the children received or lowered the quality of their home environment. It may simply be that, due to their financial situation, poor parents of children with ADHD may have little access to the resources and services that can help them and their children, a hypothesis we cannot test with the available MCS data. Another possibility, as we explain below, is that other aspects of parenting, unexplored in this study, may account for this effect.

While socio-economic disadvantage had robust associations with the emotional and behavioural outcomes of our sample, the effect of the home environment (household chaos) was nonsignificant and the impacts of parenting depended largely on the type of outcome. We think that some of the effect of household chaos, a proxy for overcrowding, may have been captured by our measure of socio-economic disadvantage that assesses overcrowding in the home objectively, or indeed by our parenting measures with which household chaos correlated more strongly. As for parenting, a poorer parent–child relationship and harsher parental discipline were predictive of more conduct problems, although neither predicted emotional symptoms. The findings for conduct problems are consistent with evidence suggesting that children and adolescents with ADHD report poorer relationships with their parents, in turn associated with externalising symptoms [28, 42, 44–46]. They are also consistent with evidence suggesting that harsh parental discipline can play an important role in the development of comorbid externalising problems in children with ADHD [11, 45, 47–52]. Parenting programmes for families with children with ADHD, therefore, that teach parents skills to improve relationships and manage behaviour may help to attenuate child externalising problems [53, 54]. As for emotional symptoms, there is some support from previous research for the null effect of similar aspects of parenting on emotional symptoms in children with ADHD [3]. We must acknowledge, however, that another possibility may simply be that other dimensions of parenting (e.g. psychological control), unexplored in this study, are more important for the emotional problems of ADHD children.

We must also acknowledge several study limitations. First, although this was a rather large sample of children with this relatively uncommon condition, statistical power was low. Second, some of the scales used had weak reliability. With a small sample size, this amount of measurement error is concerning. Third, parent reports were relied upon for most measures including the child’s emotional and conduct problems but also whether she had ever received a formal ADHD diagnosis. The use of parent reports for children’s clinical diagnoses may be subject to biases, and parents may not be the most reliable source of their children’s emotional and conduct problems. Nonetheless, it appears that, for children with ADHD, parent reports of externalising comorbid conditions are at least as reliable as teachers’ [55]. Also, there is good evidence of convergent validity for parent-reported ADHD diagnosis by a health care provider, at least in the US [56]. Fourth, and related to this, parents of children with ADHD may have a negative perception of their relationship with their child [57]. Although we had third-party observational data for the quality of emotional support, most of our parenting measures were parent-reported. Fifth, this study is correlational, and so our findings cannot be causally interpreted. For example, as we only had data on the quality of emotional support and the parent–child relationship at age 3, we could not test the (plausible) hypothesis that problem behaviour in children with ADHD was the cause rather than the outcome of low parental responsiveness. Sixth, we did not have information on any pharmacological treatment, which may alter the development (or the parental perception) of behaviour problems in children with ADHD. We did not have data on ADHD symptom severity either, which could confound the comorbidity we tried to explain in this study [58].

In conclusion (and noting the limitations above), this study demonstrated that family socio-economic disadvantage puts children with ADHD at an increased risk of developing emotional and conduct problems. One plausible explanation may be that ADHD and emotional and conduct problems have certain common aetiological factors that are more prevalent in poorer families. Importantly, it also showed that poor parent–child relationship, as well as parents’ harsh discipline practices are associated with conduct problems in children with ADHD. Nonetheless, it did not find that the association between socio-economic disadvantage and emotional and conduct problems in children with ADHD was explained by household chaos or harsh/disengaged parenting. Future studies should explore mediation by additional parenting measures, such as those indexing parenting practices rather than styles. For example, parental involvement or the home learning environment may be more relevant for children with cognitive impairments, such as children with ADHD, and thus perhaps more likely to mediate risk effects on common comorbidities in ADHD.

## Electronic supplementary material

Below is the link to the electronic supplementary material.

Supplementary material 1 (DOCX 24 kb)

## Footnotes

1 The clustering of families within wards was unaccounted for as only around 4% of the variance in children’s emotional and behavioural problems was due to MCS children’s wards.
