# Supplementary material for: The role of socio-economic disadvantage in the development of comorbid emotional and conduct problems in children with ADHD
Source: Eur Child Adolesc Psychiatry. 2017 Jan 7;26(6):723–32. doi: 10.1007/s00787-017-0940-z (PMC5446547; doi:10.1007/s00787-017-0940-z)
Supplement: Supplementary file 1 — Supplementary material 1 (DOCX 24 kb) [file 787_2017_940_MOESM1_ESM.docx]

**Random parameters**: The random parameters are between-occasion variance (variation in scores between occasions for each child), between-child intercept variance (variation in scores between children at age 7), between-child linear slope variance (variation between children in the average rate of annual change in scores), between-child quadratic slope variance (variation between children in the non-constant average rate of annual change in scores), covariance of the between-child intercept variance and the between-child linear slope variance (showing the relationship between scores at age 7 and their average rate of annual change), covariance of the between-child intercept variance and the between-child quadratic slope variance (showing the relationship between scores at age 7 and their non-constant average rate of annual change), and covariance of the between-child linear slope variance and the between-child quadratic slope variance (showing the relationship between the variances of the linear and the quadratic slopes).

**Supplementary Table 1**

Fixed and random effects on emotional and conduct problems.

|  | Model 2 | | | | |  | Model 3 | | | | |
| --- | --- | --- | --- | --- | --- | --- | --- | --- | --- | --- | --- |
|  | Emotional Problems | |  | Conduct Problems | |  | Emotional Problems | |  | Conduct Problems | |
|  | Coeff. | SE |  | Coeff. | SE |  | Coeff. | SE |  | Coeff. | SE |
|  |  |  |  | **Fixed Effects** | | | | |  |  |  |
| Age | 0.325*** | 0.042 |  | 0.363** | 0.120 |  | 0.294*** | 0.048 |  | 0.286* | 0.135 |
| Age^2^ | -0.027* | 0.014 |  | -0.028*** | 0.007 |  | -0.033 | 0.017 |  | 0.039** | 0.014 |
| Age^3^ |  |  |  | 0.031* | 0.014 |  |  |  |  | -0.024** | 0.008 |
| England Disadvantaged | 0.437 | 0.340 |  | 0.470 | 0.366 |  | -0.134 | 0.387 |  | -0.465 | 0.409 |
| England Ethnic | 1.154* | 0.516 |  | -0.124 | 0.545 |  | 1.144 | 0.812 |  | 0.550 | 0.837 |
| Wales Advantaged | 0.728 | 0.807 |  | -0.933 | 0.884 |  | 0.572 | 0.698 |  | -1.335 | 0.748 |
| Wales Disadvantaged | 0.301 | 0.416 |  | 0.297 | 0.452 |  | -0.045 | 0.414 |  | -0.529 | 0.438 |
| Scotland Advantaged | -0.232 | 0.546 |  | 0.148 | 0.599 |  | -0.330 | 0.687 |  | -0.878 | 0.720 |
| Scotland Disadvantaged | -0.910 | 0.539 |  | 0.343 | 0.590 |  | -1.326* | 0.521 |  | -0.918 | 0.551 |
| Northern Ireland Advantaged | 0.761 | 0.666 |  | -0.541 | 0.715 |  | 0.426 | 0.692 |  | -1.059 | 0.737 |
| Northern Ireland Disadvantaged | -0.491 | 0.490 |  | 0.466 | 0.526 |  | -0.591 | 0.514 |  | -0.414 | 0.539 |
| Family SED | 0.919* | 0.465 |  | 1.404** | 0.448 |  | 1.255* | 0.616 |  | 1.784*** | 0.575 |
| Family SED x Age | -0.177* | 0.090 |  | -0.281 | 0.259 |  | -0.093 | 0.109 |  | -0.334 | 0.316 |
| Family SED x Age^2^ | -0.042 | 0.031 |  | -0.057 | 0.031 |  | -0.045 | 0.040 |  | -0.082* | 0.034 |
| Family SED x Age^3^ |  |  |  |  |  |  |  |  |  | 0.018 | 0.020 |
| Female |  |  |  |  |  |  | -0.052 | 0.342 |  | -0.515 | 0.361 |
| White |  |  |  |  |  |  | -0.345 | 0.727 |  | 1.071 | 0.740 |
| Low Birthweight |  |  |  |  |  |  | 0.238 | 0.485 |  | -0.134 | 0.501 |
| BAS II naming vocabulary |  |  |  |  |  |  | 0.008 | 0.015 |  | 0.006 | 0.015 |
| BSRA-R |  |  |  |  |  |  | -0.005 | 0.011 |  | -0.026* | 0.011 |
| Maternal psychological distress |  |  |  |  |  |  | 0.123*** | 0.020 |  | 0.114*** | 0.019 |
| Mother is university educated |  |  |  |  |  |  | -0.245 | 0.433 |  | -0.160 | 0.460 |
| Two caregivers |  |  |  |  |  |  | 0.351 | 0.260 |  | 0.334 | 0.247 |
| Constant | 2.796*** | 0.306 |  | 3.347*** | 0.308 |  | 2.641* | 1.284 |  | 4.197** | 1.339 |
| **Random Effects** | | | | | | | | | | | |
| Between-child intercept variance | 2.930*** | 0.587 |  | 2.703*** | 0.515 |  | 2.930*** | 0.587 |  | 2.703*** | 0.515 |
| Between-child slope variance (age)  Between-child intercept slope variance covariance (age) | 0.060**  0.283*** | 0.020  0.080 |  | 0.053**  0.177*** | 0.017  0.066 |  | 0.060**  0.283*** | 0.020  0.080 |  | 0.053***  0.177*** | 0.017  0.066 |
| Between-child slope variance (age^2^) | 0.006* | 0.003 |  | 0.003 | 0.002 |  | 0.006* | 0.003 |  | 0.003 | 0.002 |
| Between-child intercept slope variance covariance (age^2^) | -0.091*** | 0.033 |  | -0.067*** | 0.026 |  | -0.091*** | 0.033 |  | -0.067*** | 0.026 |
| Between-child slope (age) slope (age^2^) variance covariance | -0.004 | 0.005 |  | -0.007 | 0.004 |  | -0.004 | 0.005 |  | -0.007 | 0.004 |
| Between-occasion variance | 2.063*** | 0.227 |  | 1.725*** | 0.192 |  | 2.004*** | 0.272 |  | 1.635*** | 0.220 |

*Notes.* BAS II = British Ability Scales II; BSRA-R = Bracken School Readiness Assessment Revised. **p* < .05; ***p* < .01; ***p* < .001. SED = socio-economic disadvantage.
